# Neddylation inhibitor MLN4924 suppresses growth and migration of human gastric cancer cells

**DOI:** 10.1038/srep24218

**Published:** 2016-04-11

**Authors:** Huiyin Lan, Zaiming Tang, Hongchuan Jin, Yi Sun

**Affiliations:** 1 Institute of Translational Medicine, School of Medicine, Zhejiang University, Hangzhou, Zhejiang 310029, China; 2 Laboratory of Cancer Biology, Institute of Clinical Science, Sir Run Run Shaw Hospital, School of Medicine, Zhejiang University, Hangzhou, Zhejiang 310020, China; 3 Collaborative Innovation Center for Diagnosis and Treatment of Infectious Diseases, Zhejiang University, Hangzhou, China; 4 Division of Radiation and Cancer Biology, Department of Radiation Oncology, University of Michigan, 4424B MS-1, 1301 Catherine Street, Ann Arbor, MI 48109, USA

## Abstract

MLN4924 is a recently discovered small molecule inhibitor of NEDD8-Activating Enzyme (NAE). Because cullin RING ligase (CRL), the largest family of E3 ubiquitin ligase, requires cullin neddylation for its activity, MLN4924, therefore, acts as an indirect inhibitor of CRL by blocking cullin neddylation. Given that CRLs components are up-regulated, whereas neddylation modification is over-activated in a number of human cancers, MLN4924 was found to be effective in growth suppression of cancer cells. Whether MLN4924 is effective against gastric cancer cells, however, remains elusive. Here we showed that in gastric cancer cells, MLN4924 rapidly inhibited cullin 1 neddylation and remarkably suppressed growth and survival as well as migration in a dose-and time-dependent manner. Mechanistic studies in combination with siRNA knockdown-based rescue experiments revealed that MLN4924 induced the accumulation of a number of CRL substrates, including CDT1/ORC1, p21/p27, and PHLPP1 to trigger DNA damage response and induce growth arrest at the G2/M phase, to induce senescence, as well as autophagy, respectively. MLN4924 also significantly suppressed migration by transcriptionally activating E-cadherin and repressing MMP-9. Taken together, our study suggest that neddylation modification and CRL E3 ligase are attractive gastric cancer targets, and MLN4924 might be further developed as a potent therapeutic agent for the treatment of gastric cancer.

Gastric cancer (GC) continues to be a major health problem with around 1 million new GC cases and more than 700,000 deaths annually in the world, which accounts for 10% of all cancer deaths in 2012^1^. Given that GC is often diagnosed at advanced stages when surgery and local therapies are no longer effective, survival outcomes is poor in most settings. For patients with advanced GC who developed acquired drug resistance and/or disease recurrence or metastasis following first-line chemotherapy, available therapeutic options were very limited. Novel targeted based effective therapies are in urgent need to reduce the burden of GC worldwide.

The Cullin-Ring ligases (CRLs) are the largest multiunit ubiquitin ligases that are responsible for ubiquitylation of about 20% of cellular proteins for targeted degradation^2^,^3^. CRL1, the founding member of CRLs, is also known as SCF (SKP1, cullin-1 and F-box protein) E3 ligase, which consists of scaffold protein cullin-1, adaptor protein SKP1, and substrate-recognizing F-box protein and RING component, RBX1 or RBX2/SAG^4^,^5^. Accumulated data showed that dysfunction of CRLs, particularly CRL1, is associated with many human diseases, including cancer^6^,^7^. To date, CRL1/SCF E3 ligase has been proposed as a promising druggable anti-cancer target based on the following findings: (1) CRL1/SCF E3 ligase is usually abnormally activated in many human cancers, which results in uncontrolled proliferation and genomic instability; (2) Several essential components of CRL1/SCF E3 ligase, such as RING-finger protein SAG/RBX2/ROC2, or F-box proteins SKP2 or β-TrCP, function as oncoproteins that are widely over-expressed in human cancers; (3) Inactivation of these CRL1/SCF E3 ligases or down-regulation of their oncogenic components suppressed cancer cell growth both *in vitro* and *in vivo*^8^,^9^,^10^,^11^.

The full activation of CRLs E3 ligases required cullins neddylation, a reversible modification by adding ubiquitin-like protein NEDD8 (Neural precursor cell expressed developmentally down-regulated 8) to cullins. This reaction were successively catalyzed by NEDD8-activating enzyme E1 (NAE), NEDD8-conjugating enzyme E2 (Ubc12/UBE2M or UBE2F)^12^, and NEDD8-E3 ligases^13^. MLN4924, a specific small molecule inhibitor of NAE, was recently discovered, which binds to NAE to create a covalent NEDD8-MLN4924 adduct that blocks the enzymatic activity of NAE^2^,^14^. Consequently, MLN4924 efficiently inhibits neddylation of all cullins, leading to inactivation of CRLs and accumulation of their substrates, which triggers DNA re-replication stress, DNA damage response (DDR), cell cycle arrest, apoptosis, and senescence to suppress the growth of cancer cells both *in vitro* and *in vivo*^15^,^16^,^17^,^18^,^19^,^20^,^21^. In addition, MLN4924 induces protective autophagy through inducing accumulation of SCF E3 substrates DEPTOR, a direct inhibitor of mTORC1 and the HIF1-REDD1-TSC1 axis, a negative regulatory pathway of mTORC1^21^. All these findings validate neddylation pathway and CRL1/SCF E3 ligase as promising anti-cancer targets, and further demonstrate MLN4924 as a potential drug for cancer therapy.

To date, whether and how MLN4924 performs its anticancer activity has not been fully explored, although one recent study showed a protective role of p27 in MLN4924-induced growth suppression of gastric cancer cells^22^. In the present study, we showed that MLN4924 significantly suppressed gastric cancer cell growth by blocking cullin neddylation and subsequent accumulation of a mass of CRL1/SCF E3 substrates, which trigger DNA damage response, G2-M arrest, senescence and autophagy. Furthermore, we found that MLN4924 blocks migration of gastric cancer cells which is associated transcriptional induction of E-cadherin and repression of MMP-9. Collectively, our study demonstrated that MLN4924 effectively suppressed proliferation, survival and migration of gastric cancer cells via inactivation of neddylation pathway and CRL1/SCF E3 ligase and that MLN4924 could act as a novel class of anti-cancer agent for the treatment of gastric cancer.

## Results

### RBX1 and SAG/RBX2 are over-expressed, and MLN4924 effectively inactivated cullin 1 neddylation in human gastric cancer cells

**Figure 1:**
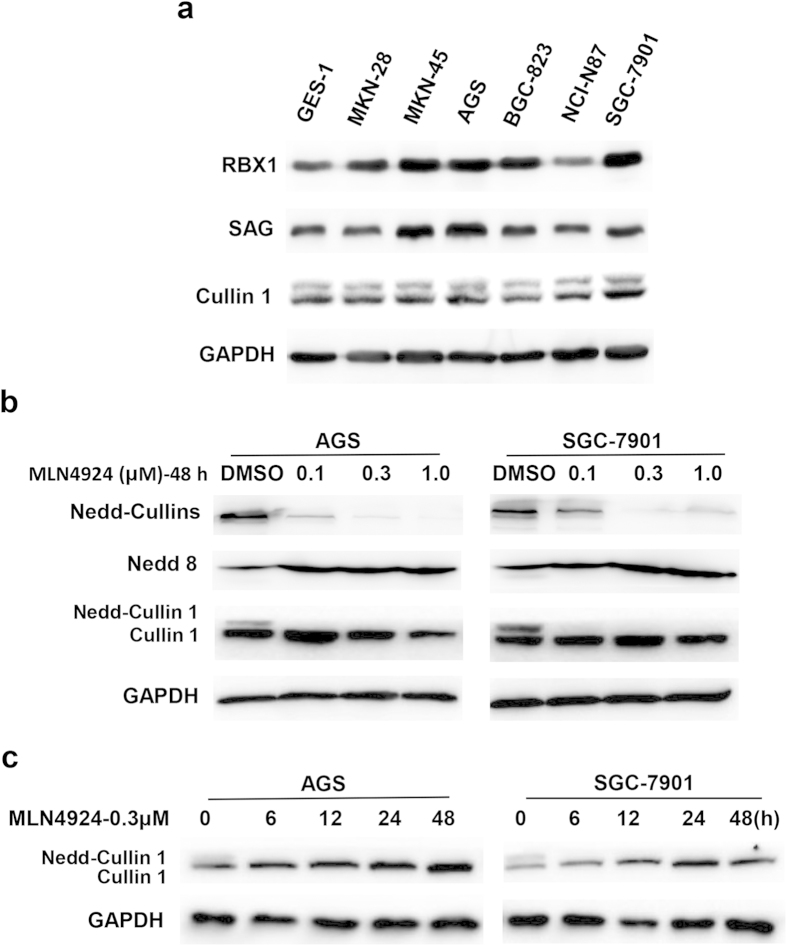
Expression of Cullin-1/RING proteins and MLN4924 effectively inhibited Cullin 1 neddylation in human gastric cancer cells. (**a**) Exponential growing cells were subjected to Western blotting analysis using antibodies against indicated proteins. (**b,c**) Cells were treated with MLN4924 at indicated doses and for indicated periods of time before being subjected to Western blot analysis using antibodies against indicated proteins. Similar results were obtained in three independent experiments.

Previous studies have shown that over-expression of RBX1 or Cullin1 was associated with poor prognosis of patients with gastric cancer^23^,^24^. To further investigate the role of CRL1/SCF in gastric cancer, we first determined the expression levels of three CRL1/SCF E3 components, RBX1, SAG/RBX2 and cullin-1 in several gastric cancer cell lines. Compared to the levels in immortalized “normal” human gastric epithelial GES-1 cells, RBX1 and SAG were over-expressed in most of gastric cancer cell lines tested, with the highest expression seen in MKN-45, AGS and SGC-7901 cells (Fig. 1a). However, expression of cullin-1 appeared to be comparable between normal and cancer cells (Fig. 1a).

We have previously shown that knockdown of either RBX1 or SAG via siRNA silencing suppressed growth of lung cancer cells by inducing apoptosis^25^,^26^. Given that there is no previous study showing any potential correlation between RBX1/SAG expression and MLN4924 sensitivity, we chose two gastric cancer lines, AGS and SGC-7901 with an over-expressed RBX1 and SAG for this study. We first determined effect of MLN4924 on cullin neddylation. Indeed, MLN4924 caused a dose-dependent inactivation of cullin neddylation, as evidenced by reduced levels of neddylated cullins, elimination of neddylated cullin-1, and accumulation of free NEDD8. As a matter of fact, MLN4924 effect was very potent, since a near complete inactivation of cullin neddylation can be seen at 0.1 μM low concentration (Fig. 1b). Furthermore, we found that at 0.3 μM, MLN4924 caused a complete inhibition of cullin-1 neddylation with 6 hr treatment (Fig. 1c). Taken together, these results showed that two RING components of CRL1, RBX1 and SAG are over-expressed in a number of gastric cancer cell lines and that MLN4924 effectively inactivates cullin neddylation.

### MLN4924 suppressed growth and survival of gastric cancer cells by inducing G2/M arrest and senescence

**Figure 2:**
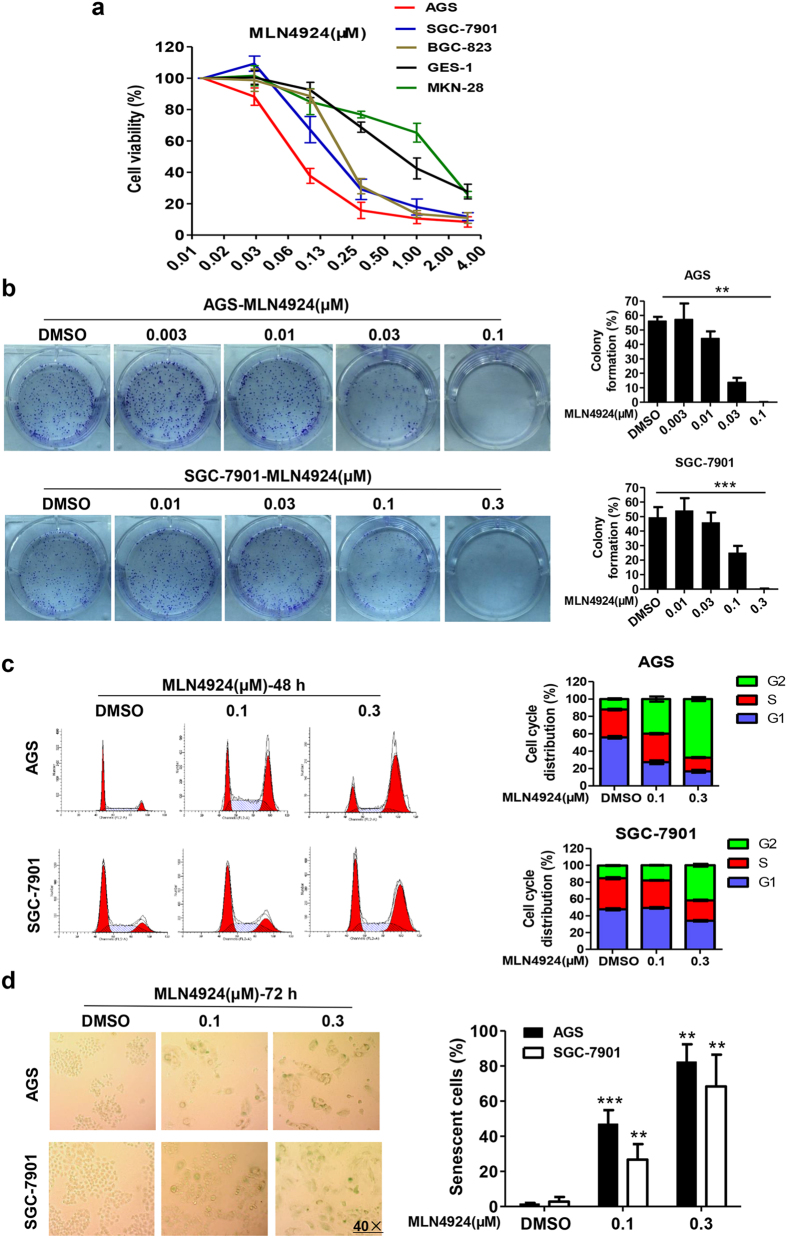
MLN4924 inhibited growth of gastric cancer cells by inducing G2-M arrest and senescence. (**a**) Cells were seeded in triplicates in 96-well plate and treated with MLN4924 at various concentrations for 72 hrs, followed by ATP-liteproliferation analysis. Shown is mean ± SEM from three independent experiments. (**b**) Cells were seeded in 60 mm dish in triplicates and subjected to MLN4924 treatment at indicated concentrations for 10 days. Shown on leftare representative dishes, and on the left are mean ± SEM from three independent experiments. ***P < 0.001, **P < 0.01, two-tailed unpaired student’s t-test. (**c,d**) Cells were treated with DMSO control or MLN4924 at indicated concentrations for 48 hrs (**c**) or 72 hrs (**d**) before subjected to FACS analysis (**c**) or SA-β-Gal staining (**d**). Shown on the left is representative FACS profiling images (**c**) or cell staining images (**d**) and on the right is mean ± SD from three independent experiments. Photos on (**d**) were taken with Leica DM4000 at 40 × amplification.

We next determined the effect of MLN4924 on cell viability and clonal survival of 4 gastric cancer cell lines in comparison with immortalized “normal” GES-1 cells. Cells were treated with various concentrations of MLN4924 for 72 hrs, followed by ATP-lite based viability assay with cellular ATP content as a readout. As shown in Fig. 2a, three cancer lines are more sensitive than GES-1 cells to MLN4924-induced growth suppression. Two RBX1/SAG high expressing lines, AGS and SGC-7901 are the most sensitive lines with an IC50 value of about 80 or 150 nM, respectively (Fig. 2a). MKN-28 cells with a low expression of RBX1/SAG similar to GES-1 cells are the most resistant. Collectively, it seemed to have a positive correlation between the sensitivity to drug and the relative expression levels of RBX1 and SAG. We further performed clonogenic survival assay using two sensitive AGS and SGC-7901 cells and found that MLN4924 caused a dosage-dependent inhibition of colony formation of with the IC50 value at 30 nM or 100 nM respectively (Fig. 2b).

To determine the nature of growth suppression, we performed FACS analysis on sensitive AGS and SGC-7901 cells, and resistant MKN-28 and GES-1 cells. MLN4924 treatment for 48 hrs caused remarkable growth arrest at the G2/M phase of cell cycle in two sensitive lines in a dose-dependent manner (Fig. 2c). A very minor G2/M arrest was seen in GES-1 cells, but no effect at all in MKN-28 cells (Fig. S1). These data indicate growth suppression is attributable, at least in part, to G2/M arrest. We further found that longer 72 hr treatment of MLN4924 induced substantial senescence, again in a dose-dependent manner (Fig. 2d), as evidenced by enlarged and flatten-shaped cells, and large population of SA-β-Gal positively stained cells, indicating a contribution of cellular senescence for growth suppression.

MLN4924 have previously shown to induce apoptosis in several cancer cell models^19^,^20^,^27^. Lack of sub-G1 peak in FACS analysis of gastric cancer cells sensitive to MLN4924 (Fig. 2c) may exclude apoptosis as a contributing factor to observed growth suppression. To further confirm this, we performed gated FACS analysis and found no increase in apoptotic cell population in both sensitive lines with 48 or 72 hr treatment of MLN4924 at 0.1 and 0.3 μM concentrations (Fig. S2a, and data not shown). Western blotting analysis showed that although BIM and NOXA, two pro-apoptotic proteins, known to be SCF substrates^26^,^28^,^29^ were accumulated, there is only a minimal increase of cleavage of caspase-3, but not PARP, two apoptotic biomarkers (Fig. S2b), suggesting growth inhibition was not due to apoptosis induction. Collectively, these data demonstrated that MLN4924 is a potent inhibitor of cell viability and clonal survival in gastric cancer cells, likely resulting from induction of G2/M cell cycle arrest (early stage) and senescence (later stage), but not apoptosis.

### MLN4924 causes accumulation of CRL substrates to induce G2 arrest and senescence, partly due to accumulation of CDT1 and p21

**Figure 3:**
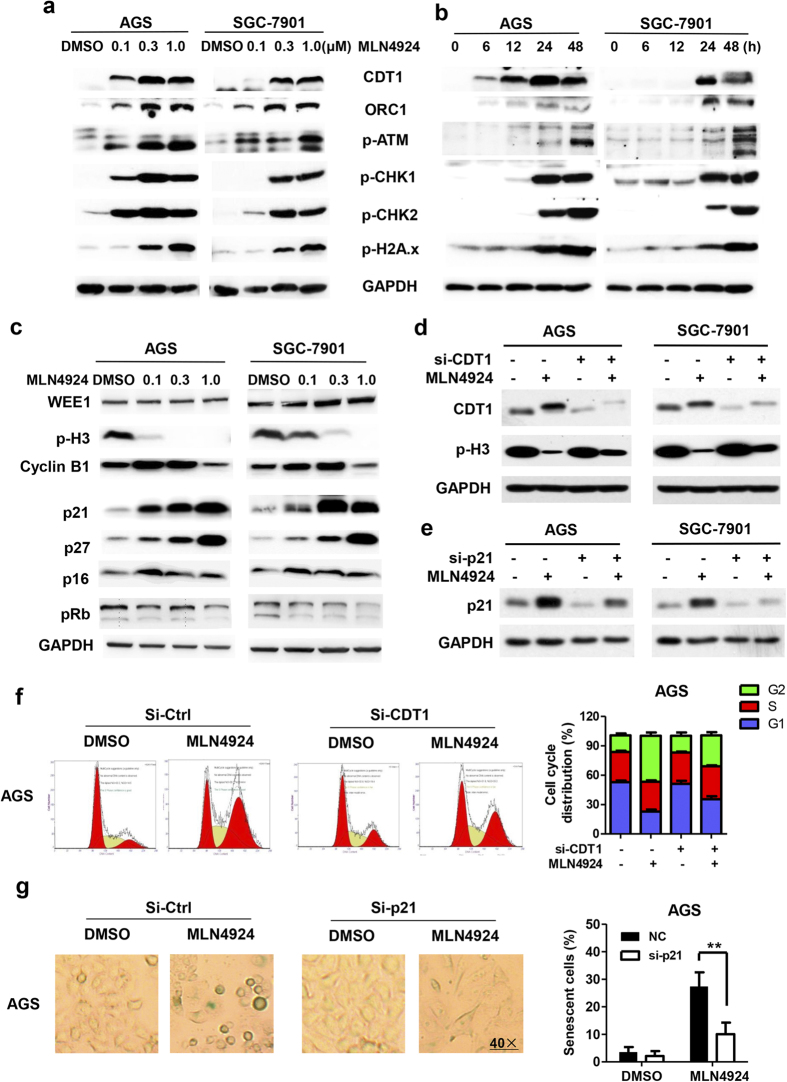
MLN4924 caused accumulation of SCF E3 substrates to trigger DNA damage response, G2/M arrest and senescence. (**a**–**c**) Cells were treated with DMSO or MLN4924 at indicated concentrations for indicated periods of time (**a**, 24 hrs, **c**, 48 hrs) before subjected to Western blot analysis using antibodies against indicated proteins. (**d**–**g**) Cells were transfected with siRNA oligonucleotides targeting CDT1 (**d,f**), or targeting p21 (**e,g**), along with scrambled control. Twenty-four hrs post transfection, cells were treated with MLN4924 for 24 hrs (**d,f**) or 48 hr (**e,g**). Cells were then split: one portion of cells was subjected for Western blotting analysis, using indicated Abs (**d,e**) and the other portion was for FACS analysis (**f**, left) or SA-β Gal staining (**g**, left). Shown on the right (**f**,**g**) is mean ± SD from three independent experiments. **P < 0.01, two-tailed unpaired student’s t-test.

We then determined molecular bases for MLN4924-induced G2/M arrest and senescence with focus on CRL substrates known to be involved in these processes, given that MLN4924 inhibits NAE and thus indirectly inactivates CRLs. Indeed, as shown in Fig. 3a,b, MLN4924 treatment of sensitive AGS and SGC-7901 cells caused a dose- and time-dependent accumulation of CDT1, a replication licensing factor and ORC1 (Origin recognition complex 1), a component of the pre-replication complex^30^,^31^. Since over-expression of CDT1 or ORC1 is known to cause DNA re-replication^32^,^33^ to trigger DNA damage response (DDR) and checkpoint controls in other cancer cell lines^34^,^35^, we measured the levels of DDR biomarkers and found again that MLN4924 caused a time- and dose-dependent increase of phosphor-ATM, pCHK1, pCHK2, and γH2AX (Fig. 3a,b). The time course experiment showed that MLN4924 caused accumulation of CDT1 and ORC1 prior to increase of these DDR biomarkers, suggesting a sequential event (Fig. 3b). We also found G2 protein Wee1 is was moderately accumulated in SGC-7901 cells, but not in AGS cells, whereas the levels of phosphor-Histone 3 and cyclin B1, two mitotic biomarkers were reduced and increased, respectively (Fig. 3c). For molecular event associated with senescence, we measured the levels of p21/p27 and pRB/p16, two well-known axes in regulation of senescence^36^, and found that MLN4924 caused accumulation of p21 and p27, two CRL substrates^37^,^38^, but not of pRB and p16 (Fig. 3c).

To further verify the causal roles of accumulated CDT1/ORC1 and p21/p27 in MLN4924-induced G2/M arrest and senescence, siRNA-based knockdown of CDT1 and p21 were performed, respectively, as the rescue experiments. Indeed, we found that, upon CDT1 knockdown, MLN4924-mediated reduction of phosphor-Histone 3 was partially reversed in both AGS and SGC-7901 cells (Fig. 3d). Furthermore, the FACS analysis revealed that MLN4924-induced G2/M arrest was also partially rescued upon CDT1 knockdown (Fig. 3f and S3a). Similarly, p21 knockdown (Fig. 3e) significantly reduced MLN4924-induced senescence in both cell lines, as evidenced by significant reduction of SA-β-Gal positive cells (Fig. 3g and S3b).Taken together, it appears that MLN4924 mainly caused cell cycle arrest at the phase of G2, but not of M, through triggering DDR via accumulated CDT1 and ORC1, and MLN4924-induced senescence is mediated by p21, but not pRB/p16, as also demonstrated in other cancer cell types^17^.

### MLN4924 induced PHLPP1 accumulation to trigger protective autophagy

**Figure 4:**
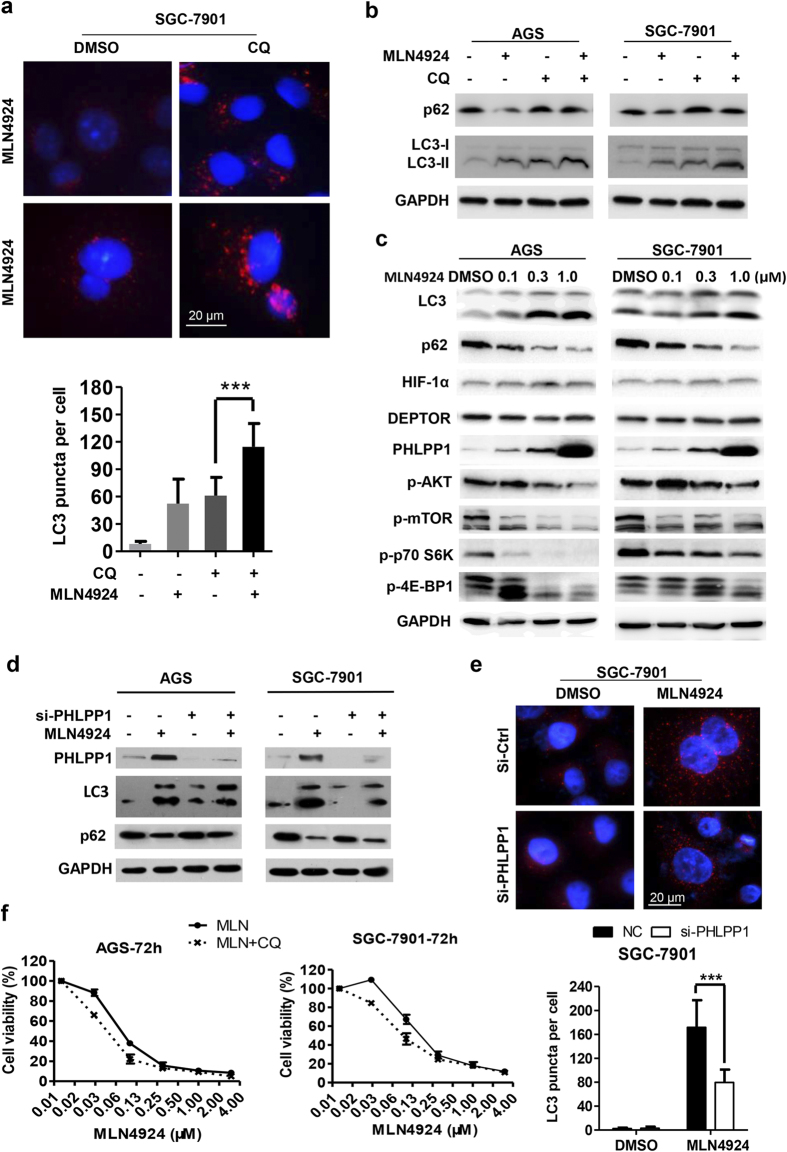
MLN4924 induced protective autophagy in gastric cancer cells. (**a**) Cells were treated with DMSO, MLN4924 (0.3 μM) or CQ (3 μM) alone or in combination for 48 hrs followed by immunofluorescence staining of LC3 and analyzed by Leica microscopy (top). The number of LC3 puncta per cell were quantified (bottom) with more than 50 cells counted. (**b,c**) Cells were treated as indicated for 48 hrs and subjected to Western blot analysis using indicated Abs. Similar results were obtained in three independent experiments. (**d,e**) Cells were transfected with siRNA oligonucleotides targeting PHLPP1, along with scrambled control siRNA before MLN4924 treatment (0.3 μM) for 72 hrs. One portion of cells was subjected for Western blotting analysis (**d**), the other portion was for immunofluorescentstaining for LC3 puncta structure (**e**, top) with quantified data shown (**e**, bottom). ***P < 0.001, two-tailed unpaired student’s t-test. (**f**) Cells were treated with various concentrations of MLN4924 alone or in combination with CQ (5 μM) for 72 hrs, followed by ATP-lite survival analysis. Shown is mean ± SEM from three independent experiments.

We have recently found that MLN4924 effectively induced autophagy in breast cancer cell lines via causing accumulation of HIF1α and DEPTOR to inactivate mTORC1^21^. In two sensitive gastric cancer lines, we found that autophagy response was dramatically stimulated upon MLN4924 treatment (Fig. 4a and S4a). To observe MLN4924-induced autophagy flux, CQ (chloroquine), a classic autophagy inhibitor, was used to block the degradation of autophagic substrates, such as p62 (SQSTM1). Compared with CQ treatment alone, MLN4924-CQ combination significantly enhanced the accumulation of autophasomes and autolysosomes, as evidenced by increased number of endogenous LC3-II positive puncta in both AGS (Fig. S4a) and SGC7901 cells (Fig. 4a). Likewise, the level of LC3-II, one of the well-established markers for autophagy activation, was elevated upon MLN4924 treatment, which was further increased when combined with CQ (Fig. 4b). Consistently, autophagic substrate p62 (SQSTM1) was degraded upon MLN4924 treatment, but accumulated by blocking autophagy with CQ (Fig. 4b). Finally, in both AGS and SGC-7901 cells, MLN4924 caused a dose-dependent increase in conversion of LC3-I to LC3-II as well as p62 degradation (Fig. 4c). Taken together, these data indicate that MLN4924 can activate autophagy in gastric cancer cells.

To define underlying molecular mechanism, we focused on upstream regulators of mTORC1, known to be substrates of CRL. We found that unlike breast cancer cells^21^, MLN4924 treatment of gastric cancer cells did not cause accumulation of HIF-1α and DEPTOR, rather the accumulation of PHLPP1 (PH domain leucine-rich repeat protein phosphatase 1)^39^ in a dose-dependent manner (Fig. 4c). Consistent with the observation that PHLPP1 is a phosphatase that inactivates AKT^40^, we found that PHLPP1 accumulation did accompany with a reduction in phosphorylation of AKT as well as mTOR, along with mTORC1 inactivation, as evidenced by reduced phosphorylation of S6K and 4E-BP1 in a dose-dependent manner (Fig. 4c). To determine whether accumulated PHLPP1 is causally related to MLN4924-induced autophagy, we knocked down PHLPP1 via siRNA and found that MLN4924-induced LC3-II elevation and p62 degradation were partially reversed (Fig. 4d). Moreover, MLN4924-induced LC3-II puncta formation in the autophagy flux was also compromised upon PHLPP1 knockdown (Fig. 4e and S4b). Collectively, these data suggested a signaling pathway in which MLN4924 triggers autophagy by causing PHLPP1 accumulation to inactivate AKT and mTORC1.

To address the role of MLN4924-induced autophagy response in growth suppression of gastric cancer cells, CQ were administrated in combination with MLN4924. After optimizing the appropriate concentration of CQ to be 5 μM for 72 h treatment, which was capable of blocking autophagy without affecting the growth of AGS/SGC-7901 cells (data not shown), we determined whether CQ could sensitize gastric cancer cells to MLN4924 by cell proliferation assay, and found that MLN-CQ combination attenuated IC50 of MLN4924 alone from about 80 nM to 40 nM in AGS cells and 150 nM to 80 nM in SGC-7901 cells, respectively (Fig. 4f). Thus, it appears that MLN4924/PHLPP1-induced autophagy was protective for gastric cancer cell growth, and the blockage of autophagy enhanced the efficacy of MLN4924.

### MLN4924 suppressed migration by up-regulating E-cadherin and down-regulating MMP-9

**Figure 5:**
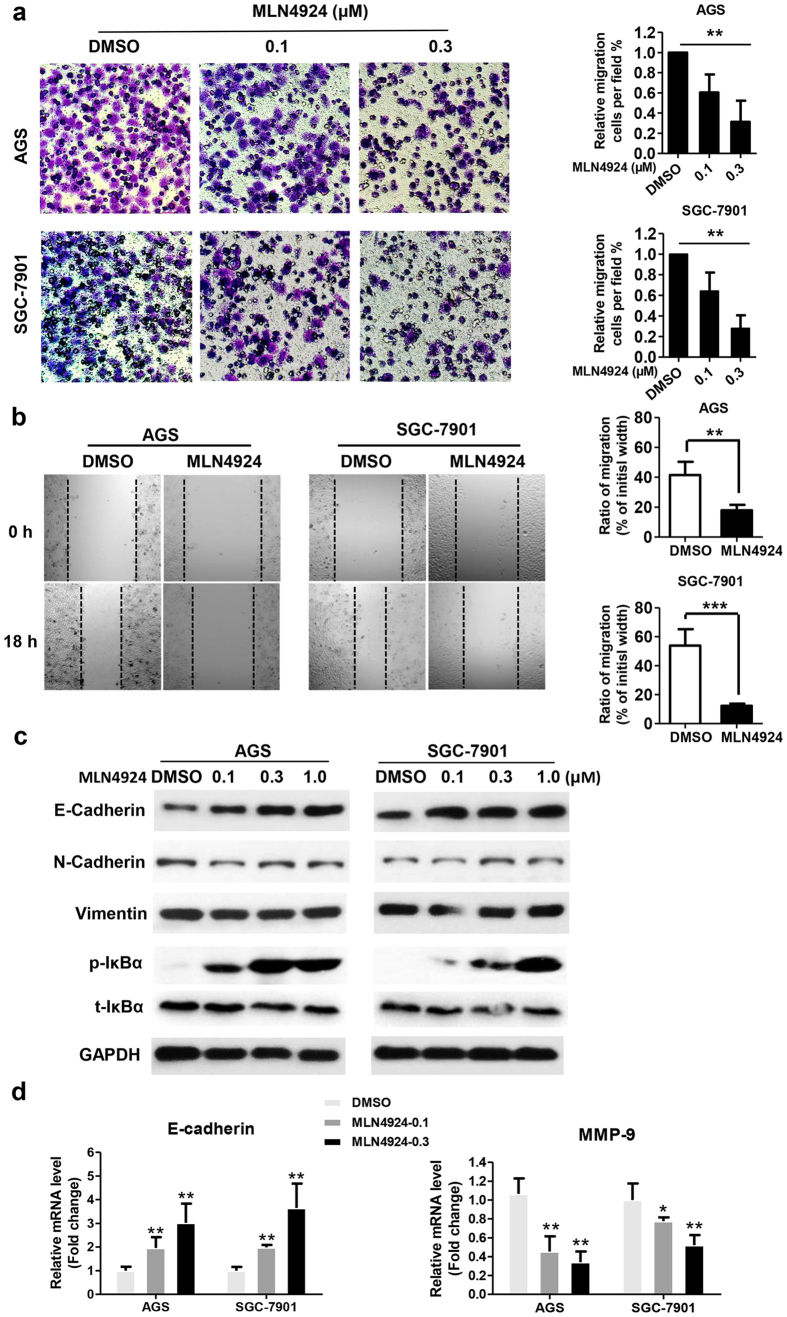
MLN4924 suppressed migration by up-regulating E-cadherin and down-regulating MMP-9. (**a**) Twenty-four hr pre-starved cells were treated with indicated concentrations of MLN4924 for 12 hr before being subjected to Transwell migration analysis. Shown are representative images (left) or mean ± SD from 200 cells per well in triplicates. **P < 0.01, one-way ANOVA statistical analysis. Similar results were obtained in three independent experiments. (**b**) Cells were treated with MLN4924 (0.1 μM) for 18 hrs before being subjected to wound-healing analysis. Shown are representative images (left) and mean ± SD from triplicates. ***P < 0.001, **P < 0.01, two-tailed unpaired student’s t-test. Similar results were obtained in three independent experiments. (**c**) Cells were treated with MLN4924 at indicated concentrations for 48 hrs and subjected to Western blot analysis using indicated Abs. (**d**) Cells were treated with MLN4924 at indicated concentrations for 48 hrs, followed by total RNA isolation and qRT-PCR analysis for E-cadherin (left) and MMP9 (right). Data were plotted after normalization and analyzed by one-way ANOVA followed by Bonferroni post hoc test using GraphPad Prism statistical programs. Shown is mean ± SD. Similar results were obtained in three independent experiments.

To explore whether MLN4924 affects migration of gastric cancer cells, we used the transwell migration and wound-healing assays as functional readouts. Indeed, MLN4924 efficiently and significantly inhibited cell migration after 12 h (or 18 h) treatment in a dosage-dependent manner in both assays (Fig. 5a,b). Importantly, the concentration and duration used for MLN4924 treatment is non-toxic to cells (data not shown), indicating MLN4924-induced migration suppression is an intrinsic effect, not the consequence of cytotoxicity.

To elucidate underlying mechanism, we focused on potential changes in the levels of biomarkers for epithelial-to-mesenchymal transition (EMT). We found that MLN4924 increased the expression of E-cadherin, an epithelial biomarker in a dosage-dependent manner in both gastric cancer cell lines, without affecting the levels of mesenchymal markers N-cadherin and Vimentin (Fig. 5c). To determine whether altered degration of E-cadherin could be responsible for its accumulation upon MLN4924, we measured protein half-life of E-cadherin by blocking new protein synthesis using cycloheximide (CHX) and found that MLN4924 had mininal, if any, effect on E-cadherin degradation (Fig. S5a,b). We next determined potential effect in E-cadherin transcription and found that MLN4924 dramatically increased E-cadherin mRNA level in a dosage-dependent manner (Fig. 5d), suggesting the transcriptional activation is accountable for its accumulation upon MLN4924 treatment.We further determined effect of MLN4924 on transcription of other EMT regulators, including N-cadherin, fibronectin and Vimentin, and found that it had minimal, if any, effect (Fig. S5c).

Given that 1) matrix metalloproteinase 9 (MMP-9) promotes cell migration^41^, 2) MMP-9 is a known target of NFκB^42^ and 3) MLN4924 inactivates NFκB by causing accumulation of IκBα (Fig. 5c), we determined potential involvement of MMP-9 in cell migration and found that MLN4924 effectively inhibited expression of MMP-9 mRNA in both gastric cancer cell lines (Fig. 5d). In contrast, we found that MLN4924 has no effect on the expression of MMP-2 mRNA (Fig. S5c), suggesting the effect is rather specific. Taken together, our results showed that MLN4924-induced suppression of cell migration is likely mediated by transcriptional upregulation of E-cadherin, and downregulation of MMP-9.

## Discussion

High mortality of gastric cancer is partly attributable to lack of effective chemotherapy and development of chemoresistance^43^. Indeed, there is no targeted therapy developed for the treatment of grastric cancer patients^44^. Recent studies have clearly shown that cullin RING ligase and neddylation pathway, which is required for its activity, are over-activated in various human cancers and targeting CRL via inhibiting neddylation pathway by a small molecule inhibitor MLN4924 is an effective anticancer approach, as demonstrated in both preclinical^7^,^10^,^13^,^18^ and clinical settings^45^,^46^. However, whether MLN4924 is effective against gastric cancer cells remains elusive. In this study, we investigated potential anti-gastric cancer activity of MLN4924 and elucidated the underlying mechanisms. We found that MLN4924 suppressed growth, survival and migration of gastric cancer cells via multiple mechanisms, acting at both transcriptional and post-translational levels. Specifically, growth suppression is mediated mainly by G2 arrest and senescence, but not apoptosis, whereas autophagy confers protection to cancer cells. Compared to previously reported in other human cancer cell models^15^,^16^,^17^,^18^,^19^,^20^,^21^, we made the following unique aspects of MLN4924 effect on gastric cancer cells.

First, the G2 arrest is unlikely mediated by Wee1, as described in a previous study using pancreatic cancer cell lines^47^, since Wee1 accumulation was minimal in gastric cancer lines. Rather, it was mediated by sequential accumulation of CDT1/ORC1 to trigger DDR and cause G2 arrest. Our rescuing experiment with CDT1 knockdown further supports this conclusion. It is worthy noting that lack of accumulation of phosphor-H3 and lack of disappearance of cyclin B1 suggests that cells were not arrested at the M phase.

Second, MLN4924 indeed induced autophagy through mTORC1 inactivation. This is, however, not mediated by accumulated HIF1α and DEPTOR, as seen in breast cancer cells^21^. Rather, it was mediated by accumulated PHLPP1, a known substrate of SCF^βTrCP ^39, which partially inhibited the AKT serine kinase activity, and mTORC phosphorylation, leading to mTORC1 inactivation to induce autophagy. Similar to what observed in liver cancer cells^20^, autophagy appears to be protective and MLN4924-induced growth suppression can be enhanced when combined with autophagy inhibitor CQ. The finding is consistent with our previous study showing that autophagy-deficient Atg5^−/−^ MEF cells were much more sensitive than autophagy-competent Atg5^+/+^ cells to MLN4924-induced growth suppression. In addition, another autophagy inhibitor Baf A1 could also enhance cell killing via blockage of autophagy^21^.

Third, MLN4924 inhibited migration of gastric cancer cells when applied at non-toxicity concentration. This appears to be mediated by transcriptional activation of E-cadherin and transcriptional repression of MMP-9. E-cadherin is the central mediator of cellular adhesion junctions and is required for the maintenance of the epithelial phenotype. E-cadherin was ubiquitinated by Hakai, a c-Cbl-like E3 ligase^48^. However, it is previously unknown whether it is subjected to neddylation regulation, which is an interesting subject for future investigation. MMP-9 is known as one of the matrix metalloproteinases (MMPs) involved in the degradation of the basement membrane in tumor invasion and metastasis, whose up-regulation has been associated with tumor aggressiveness and poor prognosis, including gastric cancer^41^,^49^. It is conceivable that repression of MMP-9 is mediated by inactivation of NFκB due to IκB accumulation, given MMP-9 is a known transcriptional target of NFκB^42^.

**Figure 6:**
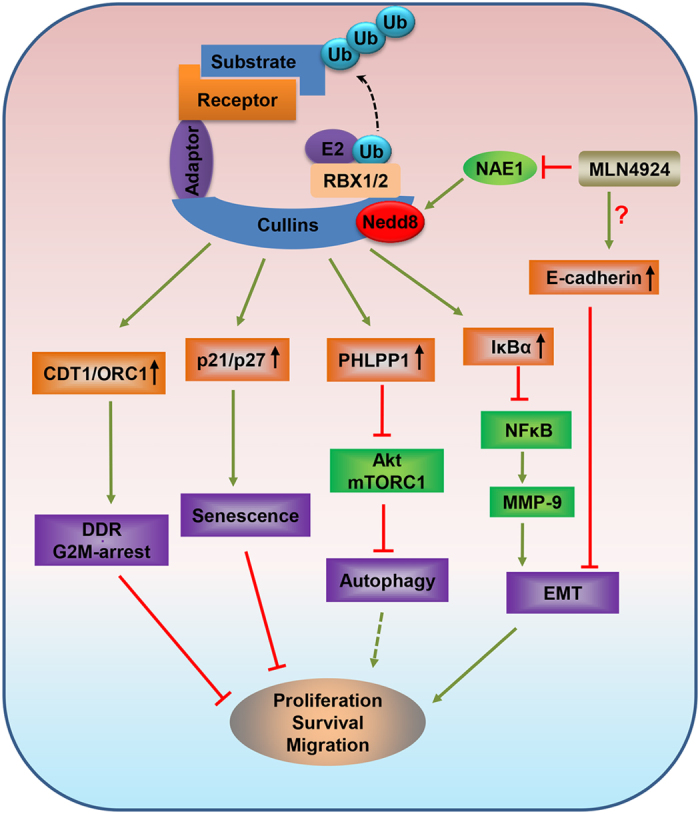
Signaling pathways that mediate MLN4924-induced growth suppression in gastric cancer cell: a working model. By inactivating NAE, MLN4924 inhibits CRL E3 ligase activity, leading to accumulation of (a) CDT1/ORC1 to trigger DNA damage response and G2/M arrest; (b) p21/p27 to induce senescence; (c) PHLPP1 to induce autophagy; (d) IκB to inhibit MMP9 and by a unknown mechanism to increase E-cadherin to block migration and invasion. Thus, CRL E3 ligase is valid target and MLN4924 is effective therapeutic agent for the treatment of gastric cancer.

In summary, our study suggested the following working model: Through inhibiting neddylation E1 NAE, MLN4924 inactivates CRL by blocking cullin neddylation, which is followed by accumulation of many CRL substrates in a cell type and context dependent manner. Accumulation of CDT1 and ORC1 would cause DNA damage and trigger DSB response leading to G2 arrest. While accumulatoin of p21/p27 induces cellular senesense, accumulation of PHLPP1 would inactivate AKT and mTORC, leading to autophagy. On the other hand, accumulation of IκBα would inactivate NFκB to cause a reduction of MMP-9 production, thus restricing migration. Finally, MLN4924 transactivates the expression of E-cadherin via a yet-to-be identified mechanism to remain cancer cells in an epithelial phenotype to prevent EMT. The net outcome of these comprehensive MLN4924 effect in gastric cancer cells is effective suppression of proliferation, survival and migration (Fig. 6). Overall, our study provides the proof-of-concept evidence for future development of neddylation inhibitors as a novel class of targeted therapy for the treatment of gastric cancer patients.

## Materials and Methods

### Cell lines and chemicals

Human gastric epithelial cell line GES-1 and 6 gastric cancer cell lines (MKN-28, MKN-45, AGS, BGC-823, NCI-N87 and SGC-7901) were obtained from American Type of Cell Collection (ATCC, Manassas, VA, USA) or RIKEN BioRe-source center (Ibaraki, Japan). GES-1, MKN-28, AGS, BGC-823, SGC-7901 and NCI-N-87 cells were cultured in RPMI 1640 medium (Invitrogen, Carsbad, CA, USA), MKN-45 cells were cultured in DMEM medium (Invitrogen), supplemented with 10% fetal bovine serum and incubated at 5% CO_2_ incubator (Thermo) at 37 °C and 95% humidity. MLN4924 was purchased from ApexBio (Houston, TX, Cat No. B1036), and was dissolved in dimethyl sulfoxide (DMSO) and stored at −20 °C. CQ (chloroquinediphosphate salt, C6628) was purchased from Sigma.

### Cell viability and clonogenic survival assay

Cells were plated in 96-well plates (3 × 10^3^ cells per well) and treated with drugs as indicated. Cell proliferation was determined using the ATP-lite Luminescence Assay kit (PerkinElmer) according to the manufacturer’s protocol. For the clonogenic survival assay, 500 cells were seeded into 6-well plates in triplicate, treated with MLN4924, autophagy inhibitor CQ alone, or in combination (MLN + CQ), followed by incubation for 10 days with drug-containing fresh medium replacement every other day. The colonies were fixed, stained and counted under an inverted microscope (Olympus, Tokyo, Japan). Colonies with 50 cells or more were counted.

### Immunoblotting

Cell lysates were quantified with the Bio-Rad protein assay kit II (Bio-Rad Laboratories, 500-0002EDU). The boiled lysates were resolved by SDS-PAGE, transferred to PVDF membranes and probed with the primary antibodies for various time, The membranes were washed with TBS-T (0.01 M TRIS-HCl Buffer, 8.8 g/L NaCl, 0.1% Tween-20), then incubated with suitable HRP-conjugated second antibodies (Dako, P016102 and P021702). Protein bands were visualized using standard chemical luminescence methodology.

The sources of primary antibodies were as follows: SAG monoclonal Ab was raised against the RING domain (AA44-113)^50^, cullin1 (Santa Cruz Biotechnology, CA, USA), RBX1, caspase-3, PARP, ORC1, CDT1, p16, p21, p27, BIM, cyclin B1,E-Cadherin, N-Cadherin, Vimentin, GAPDH, phospho-mTOR (Ser2448), phospho-Akt (Ser473), phospho-CHK1 (Ser345), phospho-CHK2 (Thr68), phospho-Histone H3 (Ser10), phospho-p70 S6 (Thr389), phospho-4E-BP1 (Thr70), phosphor-IkBα (Ser 32), IkBα and DEPTOR (Cell Signaling Technology, Denver, CO, USA), phospho-H2A.x (Ser139) (Millipore, Bedford, MA, USA), NEDD8, NOXA, PHLPP1 (Abcam, Cambridge, MA), SQSTM1/p62, LC3 (Sigma, St. Louis, MO), phospho-ATM (Ser1981) (Rockland, Limerick, PA), HIF-1α (Novus Biologicals, Littleton, CO, USA).

### FACS (fluorescence-activated cell sorting) analysis

Cells were treated with MLN4924 for 24–72 h, harvested and fixed in 70% ethanol overnight. Cells were washed twice with ice-cold phosphate buffered saline (PBS) and then stained with propidiumiodide (PI, 20 mg/ml, Sigma) solution for 30 min in the dark. The samples were then analyzed using a BD FACScan flow cytometer facility for cell cycle distributions.

Apoptosis were detected by translocation of phosphatidylserine to the cell surface using an annexin VFITC apoptosis detection kit I (BD, Becton, Dickinson and Company, US). Cells were exposed to different concentration of MLN4924, then collected and stained with FITC Annexin V and PI according to manufacturer’s instructions prior to analysis by flow cytometry.

### SA-β-Galactosidase Staining

The expression of senescence-associated β-galactosidase was determined by SA-β-Galactosidase (SA-β-Gal) staining^51^ after exposing cells to MLN4924 for 72 hrs at various concentrations.

### Imnunofluorescence staining

Cells were treated with or without drugs as indicated, fixed with cold methanol for five minutes at −20 °C. Cells were then washed three times with PBS and blocked with blocking buffer (2.5% BSA + 0.1% Triton X-100 in PBS) at room temperature for 1 h. Cells were incubated with primary antibodies at 4 °C overnight, washed five times with PBS buffer and then incubated with appropriate secondary antibodies conjugated to Alexa488 and Alexa549 (Molecular Probes) for 2 h at room temperature. DNA was stained with DAPI. Slides were examined under a Zeiss AX10 microscope system (Carl Zeiss, Germany) and images were processed with ZEN LE software (Carl Zeiss).

### Transwell migration assay

Cells were starved for 24 hours prior to the experiment, and then 2 × 10^5^ cells in 200 μL serum-free medium mixed with various concentrates of MLN4924 were added to the upper chamber of Transwell (8-μm pore) migration chambers. The lower chamber was filled with 600 μL medium containing 20% fetal bovine serum. After 24 h incubation, cell staining was performed using 0.1% crystal violet for 30 min. Non-migrated cells were carefully removed from the upper chamber filter using a cotton swab, and the cells that infiltrated the filter were counted in four random fields under microscopy.

### Wound healing assay

Cells were seeded in 12-well plate and grown to complete confluence. A straight scratch wound was made in the cell monolayer using a sterile 10 μL pipette tip. Cell debris was washed away by rinsing twice with PBS and the medium was replaced with fresh MLN4924-containing medium. The wound was photographed under a microscope at 0 and 18 hrs, and the gap between the cells was measured. The ratio of migration was calculated as follows: (ratio of migration) = [(gap width of 0 h) − (gap width of 18 h)]/(gap width: 0 h). Mean values were obtained from three independent experiments.

### siRNAs and transfection

siRNAs for CDT1, p21 and PHLPP1 were synthesized by GenePharma (Shanghai, China). The sequences of siRNAs are listed in Supplementary Table 1. Cells were transfected with siRNA duplexes (10 nM) using LipofectamineRNAiMAX transfection reagent (Life Technologies) according to the manufacturer’s instructions.

### RNA preparation and SYBR green quantitative real-time PCR

Total RNA was isolated from gastric cancer cells using TRIzol^®^ Reagent (Ambion, 15596-026, lifetechnologies, USA) according to the manufacturer’s instructions. Quantitative real-time PCR analysis was carried out using SYBR Green One-step PCR Master Mix (TAKARA, Seta 3-4-1, Japan). The mRNA expression level was determined by quantitative real-time PCR using SYBR Green Master Mix (TAKARA) and ViiA™ 7 Real-Time PCR System (Applied Biosystems, U.S). Human glyceraldehyde-3-phosphate dehydrogenase (GAPDH) was used as an internal control of RNA integrity. The sequences of the primer sets used for this study are provided in Supplementary Table 2.

### Statistical Analysis

Data with two groups were analyzed by two-tailed Student’s t-tests, and data with multiple groups were analyzed by one-way ANOVA, followed by Bonferroni post hoc test using GraphPad Prism statistical programs (GraphPad Prism, San Diego, CA). Results were expressed as mean ± SEM from three independent assays. p < 0.05 was considered statistically significant.

## Additional Information

**How to cite this article**: Lan, H. *et al*. Neddylation inhibitor MLN4924 suppresses growth and migration of human gastric cancer cells. *Sci. Rep.* **6**, 24218; doi: 10.1038/srep24218 (2016).

## Supplementary Material

Supplementary Information
