# Supplementary material for: Neddylation inhibitor MLN4924 suppresses growth and migration of human gastric cancer cells
Source: Sci Rep. 2016 Apr 11;6:24218. doi: 10.1038/srep24218 (PMC4827092; doi:10.1038/srep24218)
Supplement: Supplementary Information [file srep24218-s1.pdf]

Supplemental figure legends:

Neddylation inhibitor MLN4924 suppresses growth and migration of human gastric cancer cells

Huiyin Lan[1,2#], Zaiming Tang[1#], Hongchuan Jin[2], and Yi Sun[1,3,4*]

[1]Institute of Translational Medicine, School of Medicine, Zhejiang University, Hangzhou, Zhejiang 310029, China

[2]Laboratory of Cancer Biology, Institute of Clinical Science, Sir Run Run Shaw Hospital, School of Medicine, Zhejiang University, Hangzhou, Zhejiang 310020, China

[3]Collaborative Innovation Center for Diagnosis and Treatment of Infectious Diseases, Zhejiang University, Hangzhou, China

[4]Division of Radiation and Cancer Biology, Department of Radiation Oncology, University of Michigan, 4424B MS-1, 1301 Catherine Street, Ann Arbor, MI 48109, USA.

[#]These authors contribute equally

*Corresponding authors: Yi Sun at yisun@zju.edu.cn or sunyi@umich.edu

Figure S1. FACS profiling of human gastric cancer cells (Related to Figure 2). Cells were treated with DMSO control or MLN4924 at indicated concentrations for 48 hrs before subjected to FACS analysis. Shown on the left is representative FACS profiling images, and on the right is mean ± SD from three independent experiments.

Figure S2. MLN4924-mediated growth inhibition was not due to apoptosis induction in gastric cancer cells (Related to Figure 2)**. (a, b),** Cells were treated with DMSO or MLN4924 at indicated concentrations for 48 hrs before being subjected to FACS-apoptosis analysis (a) or Western blot analysis using antibodies against indicated proteins (b). Percentage shown in (a) is mean of triplicated samples.

Figure S3. Rescue of MLN4924-induced growth arrest and senescence by siRNA based knockdown of CDT1 and p21 in SGC-7901 cells (Related to Figure 3). Cells were treated with DMSO control or MLN4924 at indicated concentrations for 48 hrs (a) or 72 hrs (b) before being subjected to FACS analysis (a) or SA-β-Gal staining (b). Shown on the left are representative FACS profiling images (a) or cell staining images (b), and on the right is mean ± SD from three independent experiments. Photos on (b) were taken with Leica DM4000 at 40 x amplification.

Figure S4. MLN4924 induced protective autophagy in SGC-7901 cells (Related to Figure 4). **(a),** Cells were treated with DMSO, MLN4924 (0.3 μM) or CQ (3 μM) alone or in combination for 48 hrs, followed by immunofluorescence staining of LC3 and analyzed by Leica microscopy

(left). The number of LC3 puncta per cell were quantified (right) with more than 50 cells counted. **(b),** Cells were transfected with siRNA oligonucleotides targeting PHLPP1, along with scrambled control siRNA before MLN4924 treatment (0.3μM) for 72 hrs. One portion of cells was split for immunofluorescent staining for LC3 puncta structure (left) with quantified data shown (right). ***$P < 0.001$, two-tailed unpaired student's t-test.

Figure S5. Effect of MLN4924 on protein half-life and mRNA levels of EMT regulators (Related to Figure 5)**. (a, b)** Cells were treated with DMSO or MLN4924 (0.3 μM) in fresh medium (10% FBS) containing cycloheximide (CHX, 50 μg/ml) for indicated time periods and harvested for Western blot analysis using indicated Abs (a). The band density was quantified using ImageJ software and plotted (b). **(c),** Cells were treated with MLN4924 at indicated concentrations for 48 hrs, followed by total RNA isolation and qRT-PCR analysis for indicated genes. Data were plotted after normalization and analyzed by one-way ANOVA followed by Bonferroni post hoc test using GraphPad Prism statistical programs. Shown is mean ± SD from three independent experiments.

Supplement Table 1. Sequence of siRNA oligonucleotides

**Gene name**	**Sence or Antisene**	**Sequence (5'-3')**
CDT1	S	CGUGGAUGAAGUACCCGACUU
	AS	GUCGGGUACUUCAUCCACGUU
p21	S	GUGGACAGCGAGCAGCUGAUU
	AS	UCAGCUGCUCGCUGUCCACUU
PHLPP1	S	GGAAGACGCUGCUUCUGAATT
	AS	UUCAGAAGCAGCGUCUUCCTT

Supplement Table 2. Primer sequences for qRT-PCR

Gene name	F or R	Primer sequence
GAPDH	F	GGAGTCAACGGATTTGGT
	R	GTGATGGGATTTCCATTGAT
E-cadherin	F	CAGAGCCTCTGGATAGAGAACGCA
	R	GGCATTGTAGGTGTTCACATCATCGTC
MMP-9	F	CCTGGAGACCTGAGAACCAATC
	R	GATTTCGACTCTCCACGCATCT
N-cadherin	F	CAGATAGCCCGGTTTCATTTGA
	R	CAGGCTTTGATCCCTCAGGAA
Fibronectin	F	GCGAGAGTGCCCCTACTACA
	R	GTTGGTGAATCGCAGGTCA
Vimentin	F	GAACGCCAGATGCGTGAAATG
	R	CCAGAGGGAGTGAATCCAGATTA

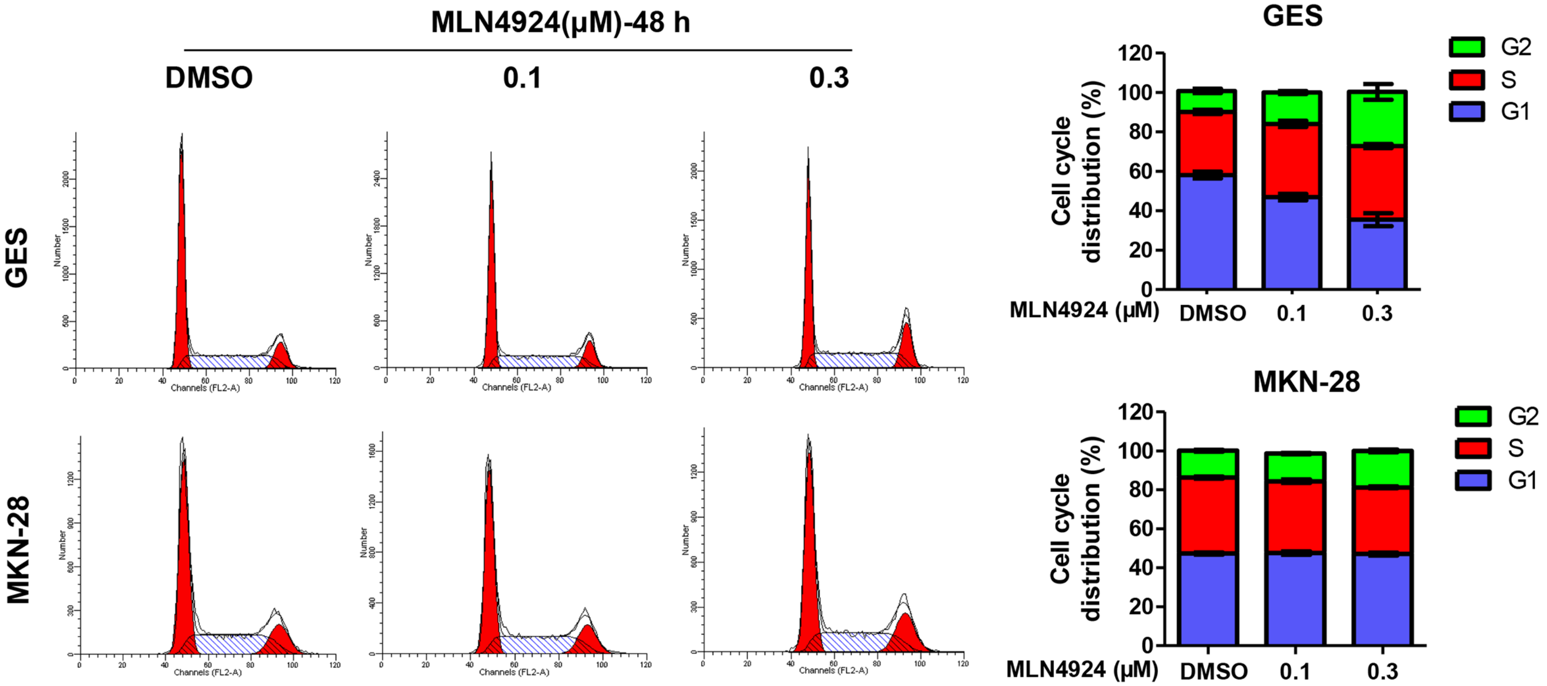

MLN4924(μM)-48 h
DMSO
0.1
0.3
GES
MKN-28
Channels (FL2-A)
Number
GES
Cell cycle distribution (%)
G2
S
G1
MLN4924 (μM)
DMSO
0.1
0.3
MKN-28
Cell cycle distribution (%)
G2
S
G1
MLN4924 (μM)
DMSO
0.1
0.3

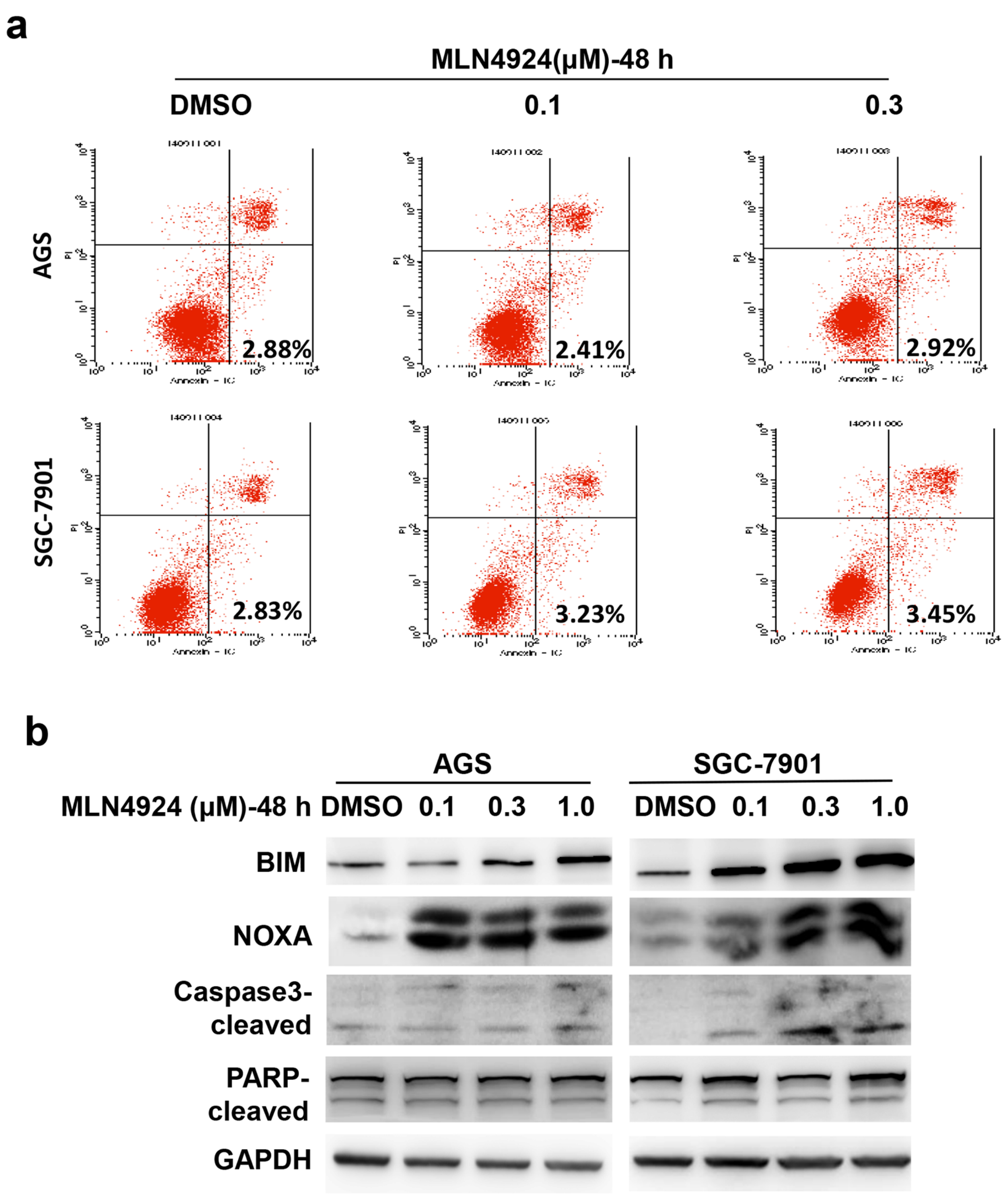
a
MLN4924(μM)-48 h
DMSO
0.1
0.3
AGS
SGC-7901
2.88%
2.41%
2.92%
2.83%
3.23%
3.45%
b
AGS
SGC-7901
MLN4924 (μM)-48 h
DMSO
0.1
0.3
1.0
BIM
NOXA
Caspase3-cleaved
PARP-cleaved
GAPDH

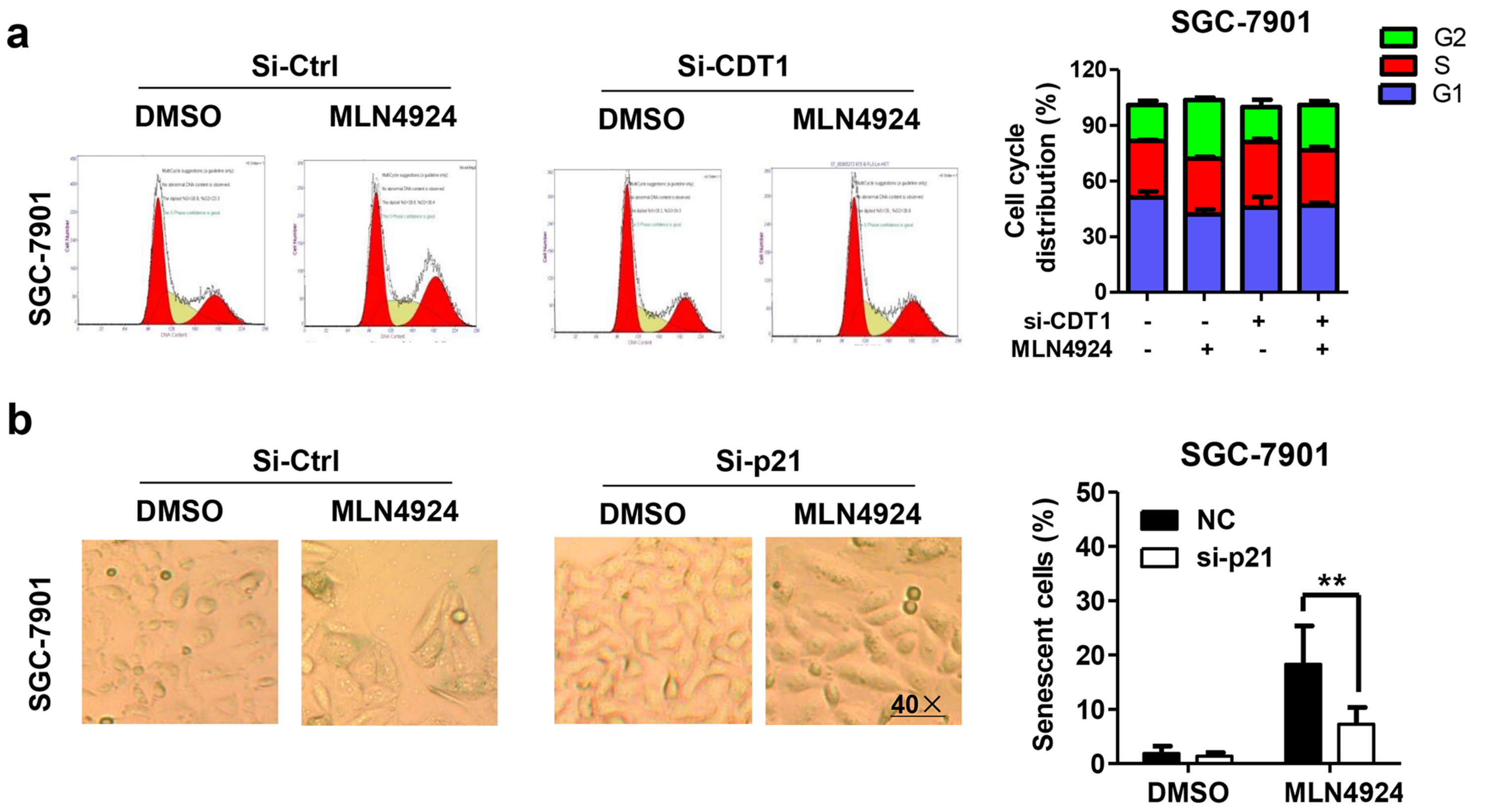
a
Si-Ctrl
Si-CDT1
DMSO
MLN4924
DMSO
MLN4924
SGC-7901
SGC-7901
G2
S
G1
Cell cycle distribution (%)
120
90
60
30
0
si-CDT1
MLN4924
b
Si-Ctrl
Si-p21
DMSO
MLN4924
DMSO
MLN4924
SGC-7901
40×
SGC-7901
NC
si-p21
Senescent cells (%)
50
40
30
20
10
0
**
DMSO
MLN4924

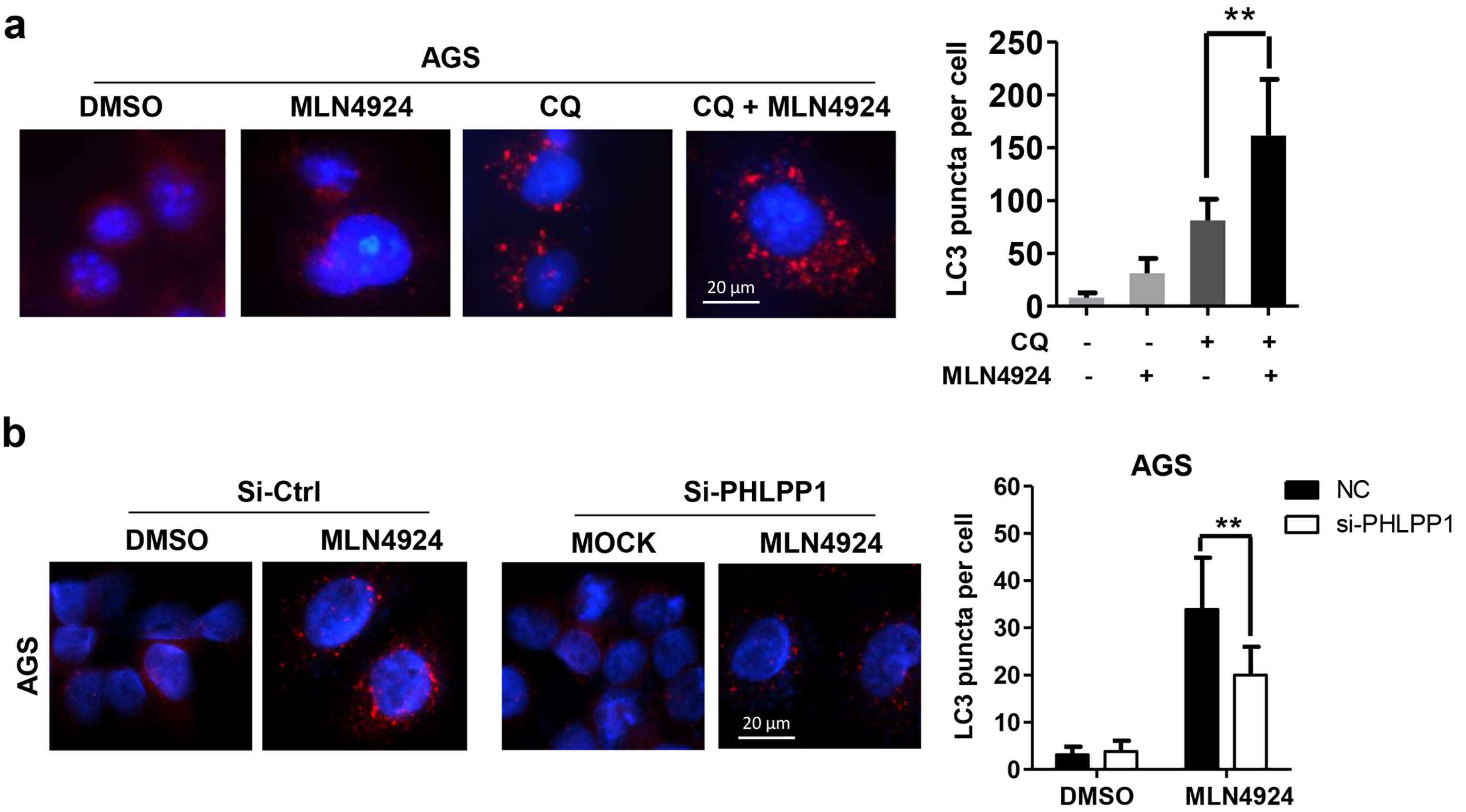

a
AGS
DMSO
MLN4924
CQ
CQ + MLN4924
20 µm
LC3 puncta per cell
250
200
150
100
50
0
**
CQ - - + +
MLN4924 - + - +
b
Si-Ctrl
DMSO
MLN4924
Si-PHLPP1
MOCK
MLN4924
AGS
20 µm
AGS
NC
si-PHLPP1
LC3 puncta per cell
60
50
40
30
20
10
0
**
DMSO
MLN4924

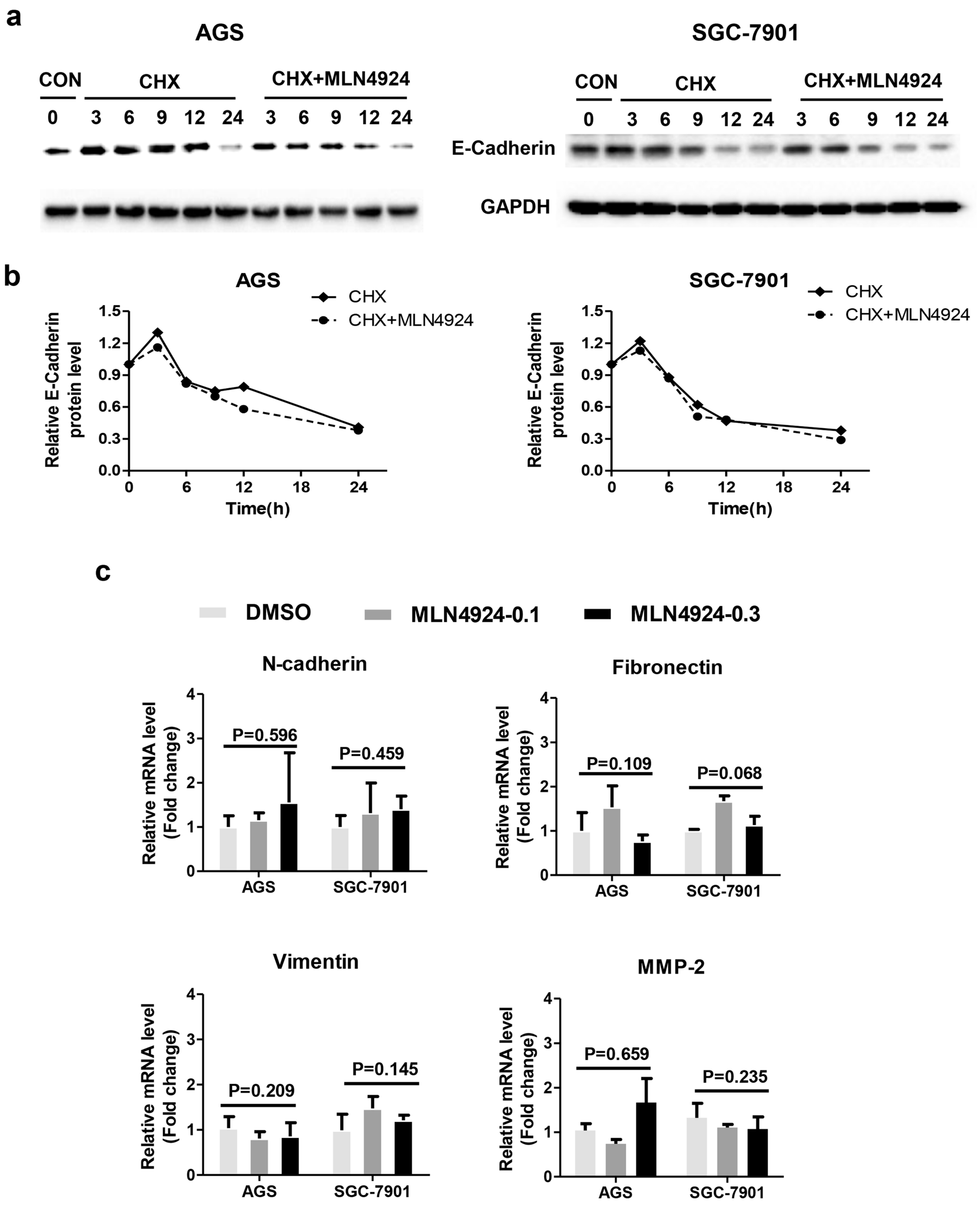

a
AGS
CON
CHX
CHX+MLN4924
0 3 6 9 12 24 3 6 9 12 24
SGC-7901
E-Cadherin
GAPDH
b
AGS
SGC-7901
CHX
CHX+MLN4924
Relative E-Cadherin protein level
Time(h)
c
DMSO
MLN4924-0.1
MLN4924-0.3
N-cadherin
P=0.596
P=0.459
Fibronectin
P=0.109
P=0.068
Vimentin
P=0.209
P=0.145
MMP-2
P=0.659
P=0.235
Relative mRNA level (Fold change)
AGS
SGC-7901
